# Supplementary material for: Activated Alpha-2 Macroglobulin Improves Insulin Response via LRP1 in Lipid-Loaded HL-1 Cardiomyocytes
Source: Int J Mol Sci. 2021 Jun 28;22(13):6915. doi: 10.3390/ijms22136915 (PMC8268138; doi:10.3390/ijms22136915)
Supplement: Supplementary file 1 [file ijms-22-06915-s001.zip › ijms-1232861-supplementary.pdf]

Activated alpha-2 Macroglobulin via LRP1 improves insulin response in lipid-loaded HL-1 cardiomyocytes.

Actis Dato Virginia, Chiabrando Gustavo A

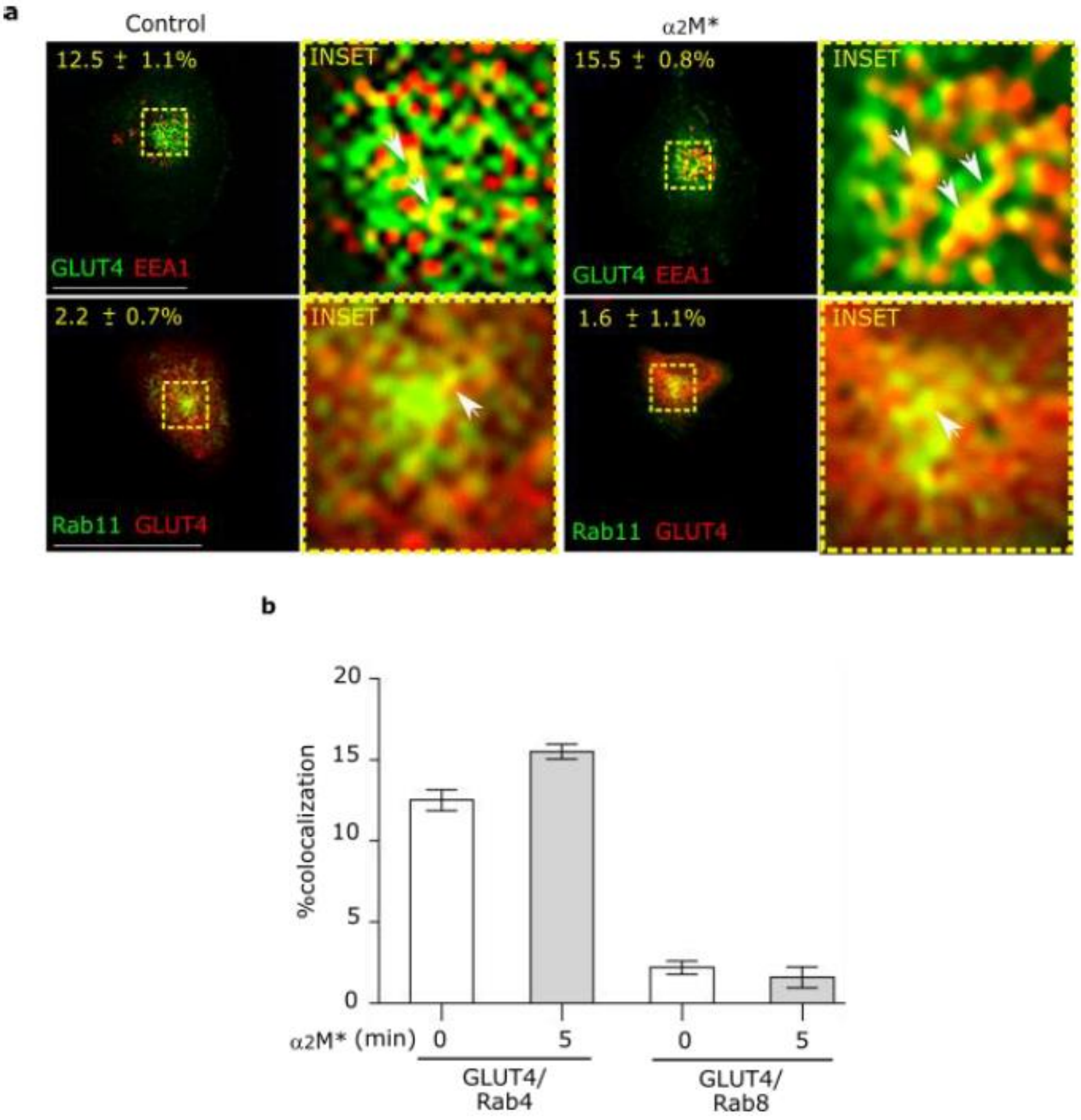

Figure S1. (**a**) Confocal microscopy in cells treated with α2M* (60nM) for 5 min at 37°C. Images show colocalization between GLUT4 and EEA1 (red) or Rab11 (green). INSET represents magnification 4X of framed regions in dotted lines. White arrowheads indicate colocalization sectors. Scale bar= 10 µm. (**b**) Quantitative analysis of colocalization between GLUT4 and different markers by Manders´coefficients expressed as mean ± SD (%).At least 20 cells per condition were analyzed (n=20).
